# Treatment and Survival Patterns of Primary Adenosquamous Carcinoma of the Liver: A Retrospective Analysis

**DOI:** 10.3389/fonc.2021.621594

**Published:** 2021-08-09

**Authors:** Qiheng Gou, Shengya Fu, Yuxin Xie, Mengni Zhang, Yali Shen

**Affiliations:** ^1^Department of Head and Neck Oncology, Cancer Center, West China Hospital, Sichuan University, Chengdu, China; ^2^Department of Abdominal Oncology, Cancer Center, West China Hospital, Sichuan University, Chengdu, China; ^3^Department of Pathology, West China Hospital, West China Medical School, Sichuan University, Chengdu, China

**Keywords:** primary adenosquamous carcinoma, liver, treatment, clinical outcome, diagnosis

## Abstract

**Background and Aims:**

Primary adenosquamous carcinoma (ASC) is a rare liver malignancy with very little data published so far. We describe the clinical characteristics of this tumor and analyze its survival pattern to improve the diagnosis and treatment.

**Materials and Methods:**

This study collected data of 15 patients with primary hepatic ASC in our hospital within 10 years (from 2009 to 2018). We analyzed the clinical characteristics, imaging data, treatment, and survival of ASC in the study. Two of these cases have been reported.

**Results:**

The common clinical symptoms of hepatic ASC are liver pain and jaundice. Laboratory examination showed that carcinoembryonic antigen (CEA) and carbohydrate antigen 19-9 (CA19-9) increased, but Alpha-FetoProtein (AFP) did not. Primary hepatic ASC is a rare subtype of intrahepatic cholangiocarcinoma (ICC) and meets the requirements of pathological diagnosis: CK20 (-), CK7 (+), CK19 (+), and p63 (+). Of the 15 patients, 11 were treated surgically, of which 3 patients received adjuvant chemotherapy. The prognosis of ASC patients is poor with a median survival time (MST) of 6 months (range: 2 to 15). The duration of MST in surgically treated patients was longer than that of nonsurgical patients (7.0 months *vs.* 3.0 months). Patients that received adjuvant chemotherapy survived longer (MST: 15 months). Patients with lymph node metastasis had a worse prognosis.

**Conclusion:**

Primary hepatic ASC is a rare malignant tumor with a poor prognosis. Radical surgery may be an effective treatment for prolonging survival. Surgical treatment combined with adjuvant therapy may further improve survival.

## Highlights

The primary ASC of the liver is rare. A total of 15 patients of primary hepatic ASC were collected within 10 years in our institute. This study shows that radical surgery may be an effective treatment for prolonging survival. Surgical treatment combined with adjuvant therapy may further improve survival.

## Introduction

Adenosquamous cell carcinoma (ASC) is a malignant tumor with adenocarcinoma (AC) and squamous cell carcinoma (SCC). Primary hepatic ASC is a rare subtype of intrahepatic cholangiocarcinoma (ICC)with very little data published so far (1). Since Pianzola and Durt first described the disease in 1971, less than 100 cases have been reported (2). It is known to be a highly aggressive tumor with a poor prognosis. The average survival time was less than 1 year (3–5). Therefore, it is of great significance to summarize more cases for the diagnosis and treatment of this rare tumor. This study reviewed the clinical data of 15 cases of hepatic ASC in our hospital and analyzed the clinical characteristics, imaging data, treatment, and survival.

## Methods and Materials

### Patients and Diagnosis

We screened the cases of primary liver cancer diagnosed in our hospital from January 2009 to December 2018. All cases included in this study were pathologically confirmed as primary hepatic ASC. We collected complete clinical data on these patients, including medical history, imaging data, pathology data, treatment, and survival. This study was approved by the Ethics Committee of West China Hospital, Sichuan University China (No.20201014).

### Treatment 

The treatment included surgical resection and nonsurgical therapy. According to the Guidelines for Diagnosis and Treatment of Primary Liver Cancer in China, surgical resection methods include hemihepatectomy, lobectomy, and segmentectomy with or without regional lymph node dissection. The nonsurgical therapy included radiotherapy, chemotherapy (oral capecitabine 1,000 mg/m2, twice daily), and targeted therapy (oral sorafenib 400 mg, twice daily).

### Statistics

The characteristics of patients were described in descriptive statics. Kaplan–Meier analysis was used to estimate overall survival (OS); log-rank testing was used to assess the difference between groups. OS was defined as the time from diagnosis to the patient’s death. P-values are considered significant if less than 0.05. All statistical analyses were performed using SPSS, version 25.0 (IBM Corp, USA).

## Results

### Patient Characteristics

In the past 10 years (January 2009 to December 2018), more than 10,000 patients with primary liver malignancies were diagnosed in our institution. Among them, only 15 patients were diagnosed as primary ASC of the liver. Eleven of them who underwent surgery were pathologically diagnosed with ASC by postoperative specimens. The diagnosis of ASC was confirmed by liver tumor biopsy in the remaining four cases without surgery. The clinical characteristics of the patients are listed in **Tables 1** and **2**. The 15 patients included 12 men and 3 women with an average age of 63.6 years (range 52 to 81).

**Table 1:** Clinicopathologic information of 15 patients.

Num	Sex	Age	First symptom	size(cm)	Postoperative pathology		Therapy			Treatment after recurrence			OS (month)
					vascular invasion	lymphatic metastasis	Surgery	Chemotherapy	Adjuvant Chemotherapy	Targeted therapy	Chemotherapy	Radiotherapy	
P1	M	65	jaundice	6.6	Y	Y	N	N	N	Y	N	N	9
P2	M	70	pyrexia /jaundice	11.89	Y	Y	Y	N	N	N	N	N	3.5
P3	F	66	bellyache	15	N	N	Y	N	N	N	N	N	9.6
P4	F	81	bellyache	6.05	N	N	Y	N	N	N	N	N	6
P5	M	56	jaundice	2.7	N	Y	Y	N	N	N	N	N	5
P6	M	75	bellyache	4	N	N	Y	N	Y	N	N	N	12
P7	F	61	bellyache	6.8	N	Y	Y	N	N	N	N	N	6
P8	M	67	bellyache	4.3	N	N	Y	N	Y	N	N	N	15
P9	M	58	bellyache	11.2	N	Y	N	Y	N	N	N	N	4
P10	M	54	bellyache	10	Y	Y	N	Y	N	N	N	N	2
P11	M	52	bellyache	4	Y	N	Y	N	N	N	N	N	5
P12	M	58	jaundice	5.8	Y	N	Y	N	Y	N	N	N	still alive
P13	M	66	bellyache	5	N	Y	Y	N	N	N	N	Y	still alive
P14	M	61	bellyache	5.8	N	N	Y	N	N	N	N	N	7
P15	M	64	bellyache	6	Y	Y	N	Y	N	N	N	N	3

**Table 2:** Clinicopathologic features of 15 cases.

Characteristics	Number	Rate
Gender		
Male	12	0.80
Female	3	0.20
Age		
<60	5	0.33
>=60	10	0.67
Primary symptom		
Abdomen pain	11	0.73
Jaundice	4	0.27
CEA (<3.4 ng/ml)		
Negative	3	0.20
Positive	12	0.80
AFP (<8 ng/ml)		
Negative	15	1.00
Positive	0	0
CA19-9 (<600 U/ml)		
<600	3	0.20
>=600	12	0.80
Tumor location		
Left	12	0.80
Right	3	0.20
Tumor size (cm)		
<5	4	0.27
>=5	11	0.73
Venous invasion		
Yes	6	0.40
No	9	0.60
Lymph nodes metastasis		
Yes	8	0.73
No	7	0.27
Intrahepatic metastasis		
Yes	1	0.07
No	14	0.93
Pathologic stage		
I	0	0
II	3	0.2
IIIA	3	0.2
IIIB	9	0.6
IV	0	0

Laboratory examination showed that the patient’s serum carcinoembryonic antigen (CEA) and carbohydrate antigen 19-9 (CA19-9) increased. The initial CA19-9 (normal <32 U/ml) was elevated in all 15 patients, of which 12 cases were significantly elevated (>=600 U/ml). Meanwhile, there were 12 patients with higher CEA (normal <3.4 ng/ml).

**Figure 1:**
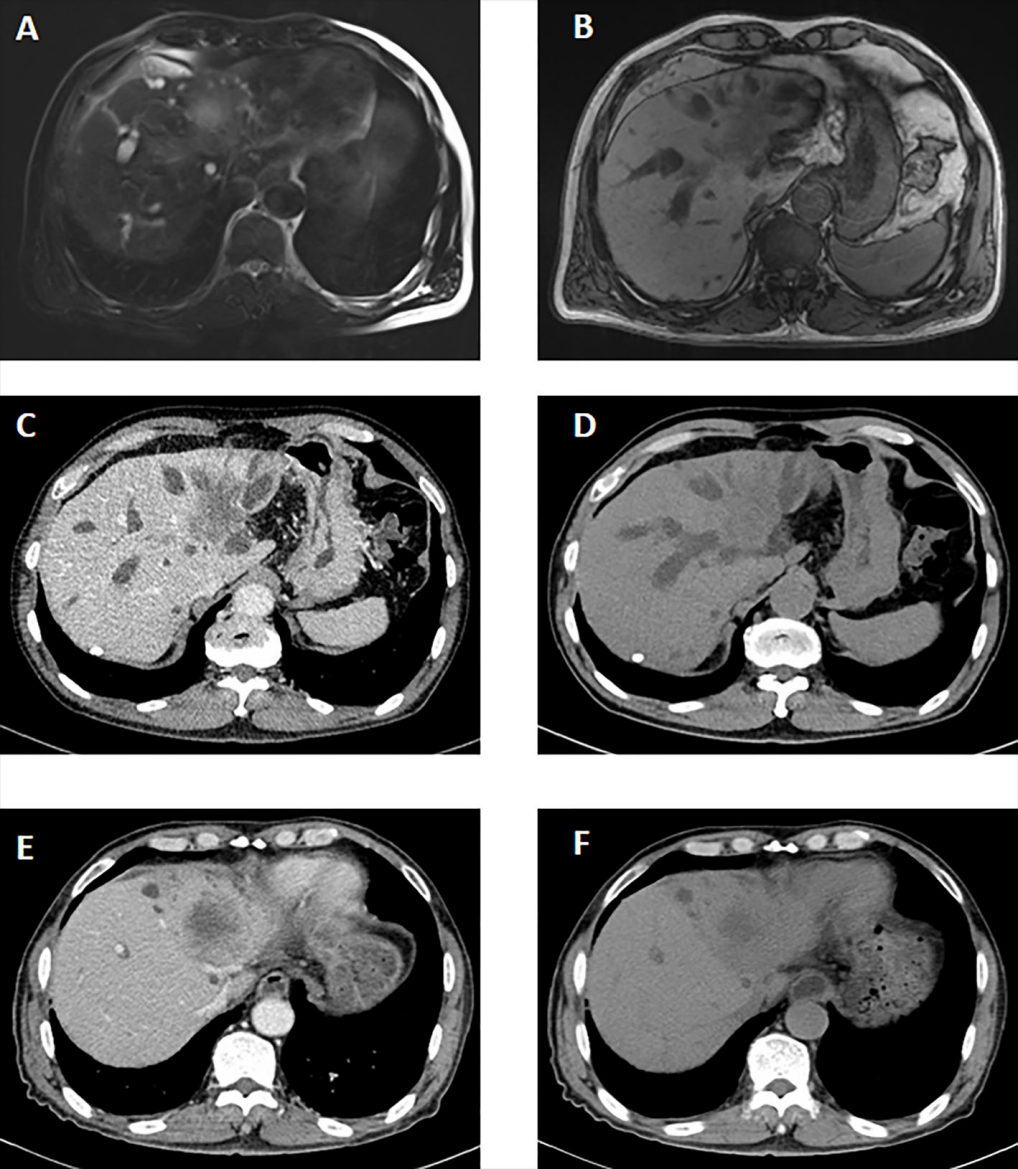
The imaging manifestations of ASC in liver. The MRI features before the therapy. **(A, B)** It showed a long T2 signal mass with heterogeneous enhancement in arterial phase and delayed enhancement in portal phase in the hilar and left lobe of liver. A slightly long T1 signal mass with an unclear boundary was seen. The CT features before the therapy. **(C, D)** A slightly low-density mass was seen in the hilum and left lobe of liver, about 6.1*3.9 cm. On contrast-enhanced scan, the lesions were slightly inhomogeneous and mainly marginal. The CT features after percutaneous transhepatic cholangial drainage (PTCD) and targeted therapy. **(E, F)** Low density mass shadow was seen in the hilus hepatis and left lobe of liver with unclear boundary. The enhancement degree of lesions after enhanced scanning was lower than that before treatment.

The imaging findings of hepatic ASC are similar to those of ICC. On the contrast-enhanced CT image, the liver tumor is irregular enhancement masses with vague margins. The liquefying necrosis in the center of the tumor is irregular without enhancement. The bile ducts around the tumor and intrahepatic bile ducts are dilated. In the MRI image, the tumor is T1-weighted low signal and T2-weighted high signal. The average tumor size measured on CT or MRI images was 7.0 cm (range: 2.7–15.0). Most patients (11/15) had a large mass (>=5 cm). According to CT image and postoperative pathological results, there were eight patients with lymph node metastasis and one with intrahepatic metastasis. At the time of diagnosis, most patients (12/ 15) were in the advanced stage (IIIA/IIIB) (**Figure 1**).

**Figure 2:**
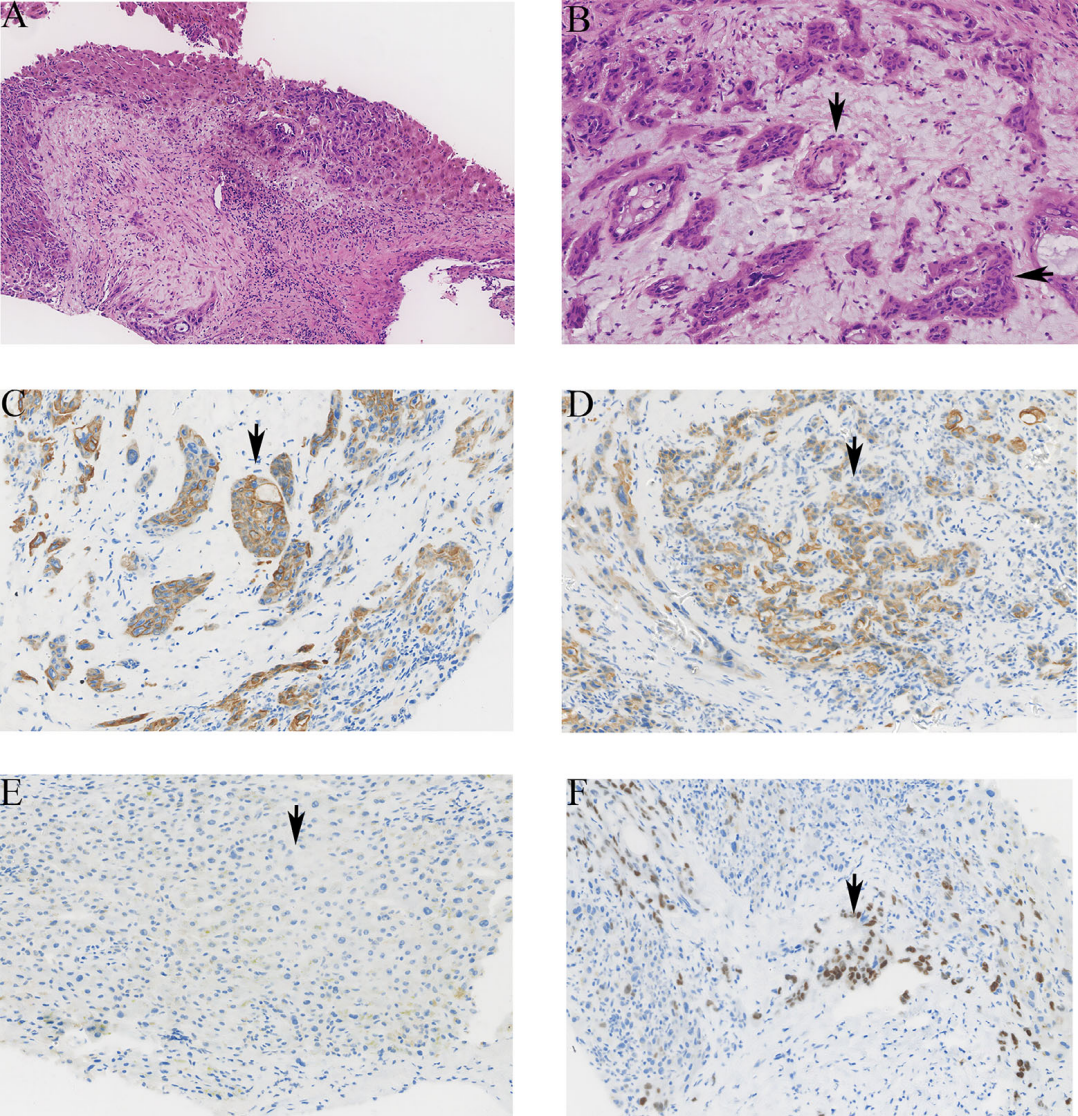
The pathological images of ASC in liver. **(A)** The tumor showed atypia, mitosis, solid nest, and tubular structure (hematoxylin and eosin stain HE ×100). **(B)** The tubular structure of the tumor is the adenocarcinoma component (vertical arrow), and the cell is nest-like, which is the squamous cell carcinoma component (horizontal arrow). **(C)** The positive staining of CK7 was brown granular in cytoplasm (×200). **(D)** The positive staining of CK19 was brown granular in cytoplasm (×200). **(E)** The negative staining of CK20 in tumor cells (×200). **(F)** The positive staining of p63 was brown granular in nucleus (×200).

Macroscopically, the tumor was gray, solid, and hard. The boundary between tumor and surrounding tissue is not clear. Satellite nodules with necrotic areas and cystic changes were seen in one case. ASC contains the components of AC and SCC. In the AC component, the tumor cells developed multifarious-sized glands and tubules with affluent fibrous stroma and generated mucin in the glandular lumen and cytoplasm. In the SCC, irregular tumor cells with eosinophilic cytoplasm and huge nuclei developed solid nests, and revealed keratin pearls and intercellular bridges. Immunohistochemical (IHC) results showed that all cases were positive for diffuse or focal CK7 and CK19. p63, a member of the p53 family, was positive in all cases. CK20 is negative in ASC cases. All cases met the pathological diagnosis requirements of ASC: CK20 (−), CK7 (+), CK19 (+), and p63 (+) (**Figure 2**).

### Outcomes

Among the 15 patients, 11 were treated surgically, of which 3 patients received adjuvant chemotherapy (oral capecitabine 1,000 mg/m^2^ twice daily). One patient with intrahepatic metastasis received targeted therapy (sorafenib 400 mg, twice daily). The remaining three patients did not receive any antitumor therapy.

**Figure 3:**
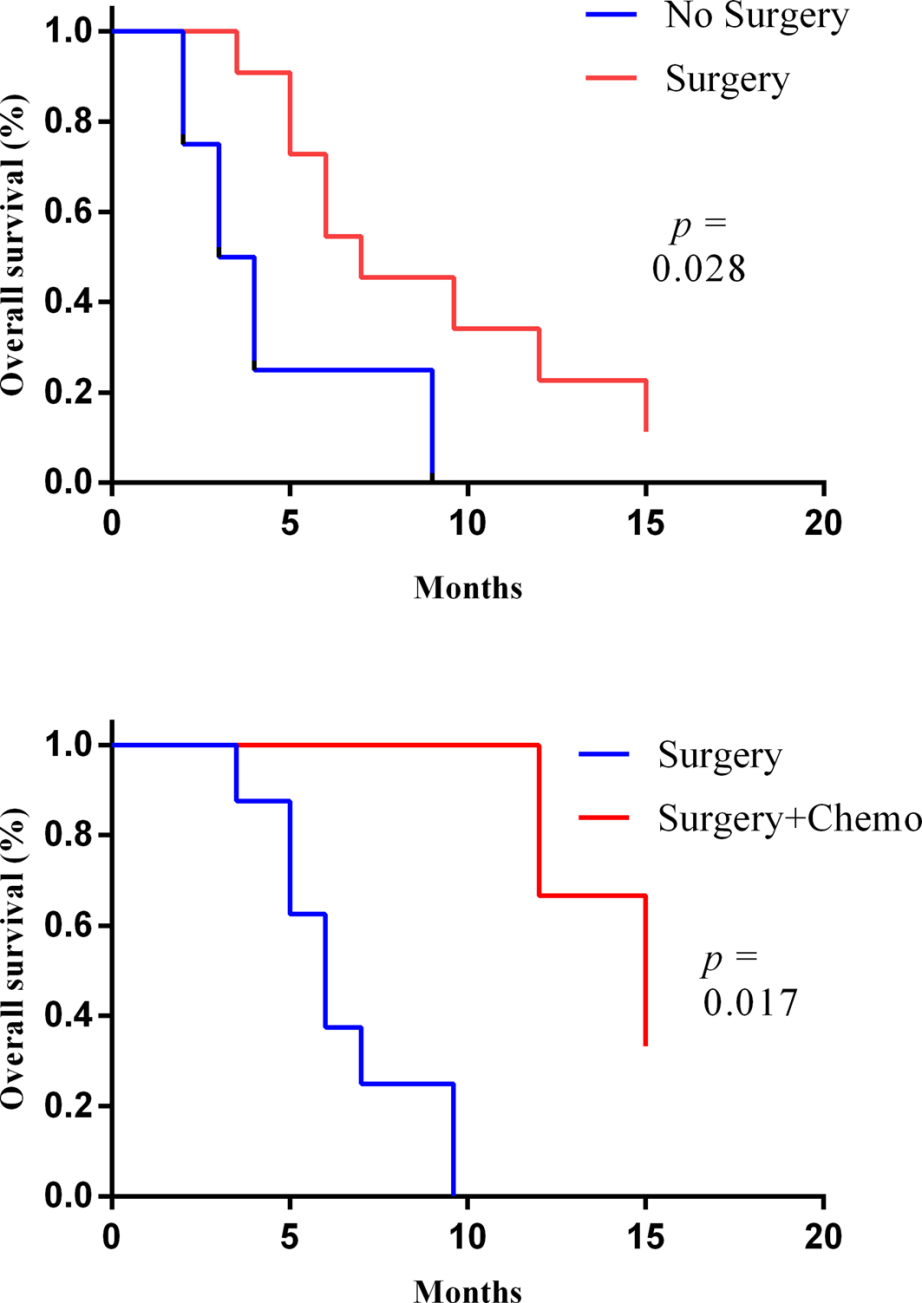
The Kaplan–Meier curves of the overall survival of patient with or without surgery (upper) and with or without adjuvant chemotherapy after surgery (lower).

During the follow-up period, 13 of the 15 patients died and 2 of them were alive. The OS of these two patients was 15 and 7 months. Then, the longest survival of patients who underwent surgery was 15 months. For all 15 patients, the median survival time (MST) was 6.0 months (range: 2–15). In the 11 patients who underwent surgery, the MST was 7 months (range: 3–15) and the 1-year survival rate was 27%. However, the median survival of the four patients without surgery was 3.0 months and none of them survived longer than 9 months. In addition, the median survival of patients with surgery combined with adjuvant chemotherapy was further prolonged than that of patients with surgery alone: 15 months *vs.* 6 months (**Figure 3**). Studies have found that patients with lymph node metastasis had a worse prognosis. Limited by the number of cases, this study did not analyze the prognostic factor.

## Discussion

As a rare subtype of ICC, few ASC data have been published. To our knowledge, it is the largest series of patients so far. It describes the clinical characteristics of this tumor and analyzes its survival pattern in 15 cases.

It is not easy to distinguish liver ASC from other primary liver tumors through clinical features including clinical symptoms, laboratory tests, and imaging examinations. ASC is mainly occuring in elderly patients with liver pain, abdominal distension, and jaundice as clinical symptoms (6), which is similar to primary hepatocellular carcinoma (HCC) or ICC. CEA and CA19-9 in the patient’s serum are increased, which is different from HCC and ICC. A previous study found that CA19-9 and maximum standardized uptake value (SUVmax) are strong prognostic factors in pancreatic ductal AC (7). However, there is no report on the prognostic correlation between PET-CT and CA19-9.

As mentioned above, it is nonspecific for imaging findings of liver ASC (e.g., marginal enhancement, delayed enhancement, lack of blood supply, and nontumor upstream bile duct dilatation) (8). Ono et al. found that 85% of liver ASC tumors were low density in CT images and 15% were iso-density. All tumors were low-signal in the MRIT1-weighted image; more than 80% of the tumors are high-signal in the T2-weighted image (9). So, the diagnosis of primary hepatic ASC depends on the pathological diagnosis. In this study, 11 patients who underwent surgery were diagnosed with ASC based on postoperative pathological results. Moreover, the remaining four patients without surgery in this study obtained the pathological diagnosis of ASC through liver tumor biopsy.

ASC contains two components of AC and SCC (10). Pathologically, the CKs are expressed in the epithelial cells of hepatocytes and bile duct (11, 12). Therefore, the expression of CKs is used to verify the cellular origin (3). In HCC, CK20 is positive and CK7 is negative. In ICC, CK20 is negative and CK7 and CK19 are positive (13). As a subtype of ICC, the diagnosis of hepatic ASC should meet the requirements of IHC findings: CK20 (-), CK7 (+), CK19 (+), and p63 (+). The pathogenesis of primary hepatic ASC remains unclear. There are two major hypotheses that chronic inflammation of bile ducts or liver cysts may lead to squamous metaplasia and malignant transformation (14, 15). Considering the IHC features, it is speculated that the ASC of the liver originated from bile duct epithelium (3, 13). However, in some reported cases, no biliary epithelial squamous metaplasia of the biliary epithelium or preexisting biliary cyst was absent within the tumor (4). In addition, ASC caused by liver cysts is rare, but three cases of liver cysts with squamous epithelium have been reported (12). Therefore, the pathogenesis of primary liver ASC is worth studying further, which may help to explore new treatments.

The prognosis of primary hepatic ASC is known to be poor. The reason may be that SCC is more invasive than AC, which is more likely to lead to lymph node metastasis or intrahepatic metastasis. There is no standard treatment for ASC. Surgical treatment combined with adjuvant therapy (including chemotherapy and radiation) may be beneficial for prolonging OS (4, 5). Regardless of the previous cases or our data, it indicates that ASC patients who underwent surgery survived longer. Maeda et al. reported that six patients with surgical resection lived for 4–8 months (3). Yeh et al. reported that the survival time of patients with resection was 6.49 months (1). Moreover, it indicated that patients with hepatic ASC who received adjuvant chemotherapy survived longer (MST, 15 months *vs.* 6 months). Demir et al. (7) and Kang et al. (16) reported that patients with hepatic ASC who received adjuvant chemotherapy survived longer, even up to 8 years. Due to the small number of samples and limited experience, the best treatment needs to be further explored in the future.

Moreover, it is worth exploring the role of novel methods in inoperable patients or patients with tumor recurrence. In this study, an inoperable patient with intrahepatic metastasis received sorafenib-targeted therapy and survived for 9 months. Some methods such as transarterial chemoembolization transcatheter arterial chemoembolization (TACE) and chemotherapy have been reported to be used in the treatment of advanced ASC. Zhang et al. reported a case with liver ASC who had tumor recurrence after surgery. This patient survived for 2 years after chemotherapy (17). Suzuki et al. reported a case of liver ASC with disease progression 3 months after surgery. The patient’s disease was stable for 15 months after TACE treatment (18). In the guidelines, immunotherapy is recommended for patients with advanced primary HCC and ICC (19). For ASC with worse prognosis, it is worth exploring the application of targeted therapy, immunotherapy, and their combination.

This study found that patients with lymph node metastasis had worse survival. Due to the limited number of cases, we did not further analyze the factors related to survival. The small sample size is a limitation of the study. For this rare disease, it is necessary to conduct multicenter research to collect more cases.

## Conclusions

Primary hepatic ASC is a rare subtype of ICC with a poor prognosis. Radical surgery may be an effective treatment for prolonging survival. Surgical treatment combined with adjuvant therapy may further improve survival. It is worth exploring the application of novel methods for liver ASC.

## Data Availability Statement

The raw data supporting the conclusions of this article will be made available by the authors, without undue reservation.

## Ethics Statement

The studies involving human participants were reviewed and approved by Ethics Committee of West China hospital, Sichuan university. The patients/participants provided their written informed consent to participate in this study. Written informed consent was obtained from the individual(s) for the publication of any potentially identifiable images or data included in this article.

## Author Contributions

Conceptualization: QG, SF, YS. Data curation: SF. Project administration: YS. Supervision: YS. Writing-original draft: QG, SF. Revision: YS. All authors contributed to the article and approved the submitted version.

## Funding

This work was supported by Youth Program of National Natural Science Foundation of China (81902723) and Sichuan science and technology support project (2019YJ0142, 2019YJ0070).

## Conflict of Interest

The authors declare that the research was conducted in the absence of any commercial or financial relationships that could be construed as a potential conflict of interest.

## Publisher’s Note

All claims expressed in this article are solely those of the authors and do not necessarily represent those of their affiliated organizations, or those of the publisher, the editors and the reviewers. Any product that may be evaluated in this article, or claim that may be made by its manufacturer, is not guaranteed or endorsed by the publisher.
